# Developmental Dynamics of Radial Vulnerability in the Cerebral Compartments in Preterm Infants and Neonates

**DOI:** 10.3389/fneur.2014.00139

**Published:** 2014-07-29

**Authors:** Ivica Kostović, Mirna Kostović-Srzentić, Vesna Benjak, Nataša Jovanov-Milošević, Milan Radoš

**Affiliations:** ^1^Croatian Institute for Brain Research, University of Zagreb School of Medicine, Zagreb, Croatia; ^2^Department of Health Psychology, University of Applied Health Sciences, Zagreb, Croatia; ^3^Department of Pediatrics, Clinical Hospital Center Zagreb, University of Zagreb School of Medicine, Zagreb, Croatia

**Keywords:** white matter injury, subplate, transient cerebral compartments, radial vulnerability, preterm infants, corridors of axonal growth

## Abstract

The developmental vulnerability of different classes of axonal pathways in preterm white matter is not known. We propose that laminar compartments of the developing cerebral wall serve as spatial framework for axonal growth and evaluate potential of anatomical landmarks for understanding reorganization of the cerebral wall after perinatal lesions. The 3-T MRI (*in vivo*) and histological analysis were performed in a series of cases ranging from 22 postconceptional weeks to 3 years. For the follow-up scans, three groups of children (control, normotypic, and preterms with lesions) were examined at the term equivalent age and after the first year of life. MRI and histological abnormalities were analyzed in the following compartments: (a) periventricular, with periventricular fiber system; (b) intermediate, with periventricular crossroads, sagittal strata, and centrum semiovale; (c) superficial, composed of gyral white matter, subplate, and cortical plate. Vulnerability of thalamocortical pathways within the crossroads and sagittal strata seems to be characteristic for early preterms, while vulnerability of long association pathways in the centrum semiovale seems to be predominant feature of late preterms. The structural indicator of the lesion of the long association pathways is the loss of delineation between centrum semiovale and subplate remnant, which is possible substrate of the diffuse periventricular leukomalacia. The enhanced difference in MR signal intensity of centrum semiovale and subplate remnant, observed in damaged children after first year, we interpret as structural plasticity of intact short cortico-cortical fibers, which grow postnatally through U-zones and enter the cortex through the subplate remnant. Our findings indicate that radial distribution of MRI signal abnormalities in the cerebral compartments may be related to lesion of different classes of axonal pathways and have prognostic value for predicting the likely outcome of prenatal and perinatal lesions.

## Introduction

The process of complex growth of white matter tracts (outgrowth of axons, pathway finding, decision making, axonal guidance, waiting periods, target selection, and in growth in the cortical plate) (1–8) occur within transient cellular compartments of the fetal cerebral wall (9, 10) and different segments of the fetal white matter (11, 12). Other neurogenetic events (proliferation, migration, molecular specification, differentiation of dendrites, synaptogenesis, gliogenesis, myelination, and cell death) also take place within the same transient laminar compartments (9, 10, 13, 14). Thus, laminar compartments provide a framework for various cellular interactions important for axonal growth and formation of axonal trajectories. In humans, the process of growth and target finding of long projection and associative axonal pathways occurs predominantly during the second half of gestation (3, 8, 10, 12, 15–17). During this period, there is sequential and partially overlapping growth of thalamocortical, commissural, and associative pathways within transient laminar compartments and sagittal axonal strata (18). Although there is significant overlap in growth of different classes of axonal pathways, there is a period of an increased growth rate for every class of afferents (3, 4, 19–22). For example, the period between 22 and 26 postconceptional weeks (PCW) is essential for terminal phases of growth of massive thalamocortical pathways and their relocation from the subplate into the cortical plate (3, 10, 15, 16). The period around 28 PCW is characterized by simultaneous growth of callosal and long associative pathways within the subplate (4, 8, 12, 23, 24). The period after 34 PCW is dominated by growth of long associative cortical pathways in parallel with the formation of secondary cortical gyri (4, 8, 19, 24–29).

As demonstrated previously, axons are more vulnerable to hypoxia ischemia and other pathogenetic factors such as periventricular hemorrhages during the period of intensive growth (6, 30–39). Therefore, transient compartments that predominantly contain growing axonal pathways (fetal white matter) are the most vulnerable cellular compartments in the preterm brain (36, 37, 40). The increased vulnerability during increased growth rate of axonal pathways is consistent with an extensive body of evidence showing that white matter injury is predominant pathology during the early third trimester (30, 31, 41–45). However, a recent review (30) suggested that, during preterm and term period, both white matter and neuronal “gray” matter are vulnerable to etiological factors, such as hypoxia ischemia. Other studies provided further evidence on the involvement of cortical “gray” in the injury of the preterm brain (46–50). In this context, the most intriguing seems to be the vulnerability of the subplate, which is the site of the earliest synaptic cortical activity and the most prominent compartment of the cerebral wall in the preterm infant. The subplate contains growing axons, postmigratory neurons, synapses, and glia (23) and is prospective target for hypoxia ischemia (30, 31, 51–53).

The presence of well defined laminar architecture of cerebral wall (23, 54) as well as defined fetal white matter segments in late fetuses (11, 18) offers a unique opportunity to study spatial parameters of selective vulnerability of different, radially arranged cerebral compartments and related growing axonal pathways. Modern imaging studies using both conventional and diffusion techniques open new vistas in study of lesions of different compartments of cerebral wall (12, 25, 26, 28, 30, 55–58). It was proposed that prenatal lesions of developmentally important transient cellular compartments imply subsequent developmental reorganization of the cerebral cortex (31, 34, 51, 53, 59–62). The imaging studies have shown that pathologies seen after ischemia and hemorrhage show differences, which partially depend on the depth location within the different segments of the cerebral white matter (11, 12, 28, 31, 36, 42, 44, 53, 63, 64). However, we still lack a detailed knowledge on the vulnerability of different classes of axonal pathways within the laminar compartments and on the vulnerability of different segments of the fetal white matter along the radial axis of the cerebral wall. Thus, developmental vulnerability of modulatory, projection, commissural, long and short association pathways, and intracortical fibers, their topographical distribution, and role in developmental reorganization and structural MR correlates remain poorly understood. In addition, the correlation with disturbances of other developmental events (proliferation, migration, synaptogenesis, dendritogenesis, myelination, and cell death) remains largely unknown.

The first step in the analysis of vulnerability of transient cerebral compartments and related cell classes is their histological delineation and elucidation of their developmental history. Based on our preview studies on laminar organization and developmental reorganization of fibers, cells, and extra-cellular matrix (ECM) in fetal and infant brain, we can reconstruct location of different classes of afferent axonal pathways within compartments of the cerebral wall (3, 8, 10, 15, 16, 23, 65–67). We found that deep, periventricular segments contain identifiable classes of axonal pathways (65). Using similar “segmental” topographical approach, we delineated axonal pathways in more superficial compartments of the cerebral wall (66). We propose that transient cerebral compartments serve as important spatial corridors for growth of different classes of axonal pathways. Therefore, we designate these transient cerebral compartments as “corridors of axonal growth.”

The objectives of the present study were: (1) to define anatomical and developmental relationships between cerebral compartments and major axonal pathways and (2) to use this data for study of laminar location and extent of structural cerebral lesions in preterm infants at birth and during early postnatal life. We rely on structural criteria and parameters developed during our long-term study of normal and damaged cortex (54, 66).

The specific aims of this study are: (a) to define anatomically periventricular, intermediate, and superficial cerebral compartments, to identify incorporated classes of axonal pathways and to describe laminar landmarks for typical lesions in the preterm brain (question: where in the cerebral wall?); (b) to show the extent and characteristics of MR signal abnormalities in different cortical compartments and white matter segments at birth and in the subsequent longitudinal MR structural follow-up until the third year of life (question: how do cerebral compartments develop after lesion?); (c) to elucidate whether there are differences in structural abnormalities after the lesion in early versus late preterms, with special consideration of the subplate zone (question: when?).

The idea behind this approach is to determine whether analysis of structural abnormalities of laminar compartments and white matter segments along radial axis (from ventricle to pia) may reveal selective time-dependent and laminar-dependent radial vulnerability of the different classes of axonal pathways preterm brain (question: which pathways are lesioned in the white matter injury?). We expect that our findings will contribute to better classification and scoring of white matter injuries in preterm infant.

## Materials and Methods

For histological delineation of cerebral compartments and white matter segments, we used different fibrillar, cellular, and ECM markers on post-mortem human brains (age range 22–44 PCW) from our large and versatile Zagreb collection. For the analysis of the specimens with pathological changes, we have used same techniques as applied for normal brains in our previous studies. The details on histological, histochemical, and immunocytochemical techniques as well as selections of antibodies were described in details in our previous papers (65, 66). *In vivo* MRI examination was conducted using a set of MRI sequences, as described previously (65, 66) on three groups of children (Table 1).

**Table 1:** **Groups of children included in histological (*in vitro*) and MRI (*in vivo*) study with classification of lesion on MRI according to SCPE grading system**.

Groups	Methods	Type of lesion	Number of cases		MRI1	MRI2
			Unilateral	Bilateral		
Premature and term children with pathology	*In vivo* MRI (23–42 PCW)	Non-cystic periventricular leukomalacia (27–30 PCW)	2	8	+	+
		Cystic periventricular leukomalacia (27–33 PCW)	2	2	+	+
		Intraventricular/periventricular hemorrhage (23–39 PCW)	1	4	+	+
		Moderate basal ganglia/thalamus/cortex lesion (40–42 PCW)	0	2	+	+
	*In vitro* histology (22–44 PCW)		7		
Normotypic prematures	*In vivo* MRI (24–31 PCW)		11		+	+
	*In vitro* histology (26–34 PCW)		12		
Normal term children	*In vivo* MRI (39–41 PCW)		4		+	−
	*In vitro* histology (39–41 PCW)		2		

*Number in parenthesis refers to PCW age range at birth. First MRI exam (MRI1) was performed at term or term equivalent age and the second MRI (MRI2) exam was performed during period between first and third years*.

The first group, consisting of 21 patients (age range at birth: 23–42 PCW), was selected from a cohort of 152 children included in another longitudinal study. The exclusion criteria were: the presence of developmental anomalies, higher-grade hydrocephalus, and massive infarctions. The inclusion criteria were: the presence of other structural lesion (visible on MRI scans) related to perinatal pathology. The severity of these lesions was graded according to the surveillance of cerebral palsy in Europe (SCPE) classification system (68, 69) as follows: non-cystic periventricular leukomalacia (PVL) (two unilateral and eight bilateral cases); cystic PVL (two unilateral and two bilateral cases); intraventricular or periventricular hemorrhage (one unilateral and four bilateral cases); and two patients with moderate basal ganglia/thalamus and cortex lesions (Table 1). All children in this group also had neurological disorders of different levels as revealed on clinical exams and SNAPII/SNAPPEII scores (70). The second group included 11 prematurely born babies (age range: 24–31 PCW), who had no signs of neurological disorders and had normal brain morphology, as independently assessed by two neuroradiologists. This group of children was regarded as “normotypic.” Both groups of prematurely born children underwent longitudinal MRI exams, the first at the term equivalent age, and the second during the period between the first and third postnatal year.

The third group was composed of four normal children born at term, who were scanned during the neonatal period (due to the extracranial indication), and had neither notable brain pathology nor any signs of neurological disorders. This group of newborns was regarded as normal. In each case, the parental consent for MRI scanning was obtained and all examinations were controlled and approved by the Institutional Review Board of the University of Zagreb School of Medicine. Sampling of the tissue for the *in vitro* experiments was performed in accordance with the Declaration of Helsinki and also was approved by the Institutional Review Board of the University of Zagreb School of Medicine. All MRI data were evaluated by two independent observers (Milan Radoš and Ivica Kostović), while histological sections were analyzed by the first author. Clinical data and testing were provided by neuropediatrician and psychologist.

For delineation of transient cerebral compartments, we use generally accepted classification (9) while the classification of crossroads and other white matter segments is used as defined previously (11, 18, 66).

Previously described MRI abnormalities at term (changes in signal intensity, loss or enhancement of borders between compartments, cysts, patchy hyperintensities, scars, periventricular hemorrhages, atrophy of white matter segments, ventriculomegalia) (11, 18, 30, 31, 35, 42, 53, 63, 64, 66, 71–73) were precisely located in one of cerebral compartments described in our previous study (66). For the purpose of the present study, we have divided cerebral compartments in to deep (periventricular), intermediate, and distal (superficial) compartments (see Table 2).

**Table 2:** **Compartmental organization of the brain with related axonal pathways**.

Deep (periventricular) compartment	Corpus callosum – *(segment I)*
	Fronto-occipital fascicle (FOF) – (*segment I*)
	Cortico-striatal fibers (Muratoff’s fascicle) – (*segment I*)
	Fronto-pontine pathways – (*segment I*)
Intermediate compartment	Crossroads of projection pathways – (*segment II*)
	– Thalamocortical fibers
	– Cortico-fugal fibers
	– Callosal radiation
	– Associative sagittal fibers
	Sagittal axonal strata – (*segment II*)
	– Thalamocortical pathways
	– Basal forebrain cholinergic afferents
	– Cortico-cortical associative fiber system
	Centrum semiovale – (*segment III*)
	– Long associative fiber system
	– Projection fibers
Superficial compartment	Gyral white matter – (*segment IV*)
	– Short cortico-cortical fibers
	– U-fibers
	Subplate/subplate remnant
	– Growing front of all afferent pathways
	– Short cortico-cortical fibers
	Intracortical fibers – (*segment V*)

*Axonal pathways are divided in segments (I–V) according to Von Monakow. Table is complementary to Figure 6*.

The analysis of histological sections stained with histochemical (AChE histochemistry) and immunohistochemical methods (fibrillar staining) and structural post-mortem MR images revealed that these compartments are arranged in radial direction from ventricular (deep) to the pial surface (superficial) (9, 54).

**Figure 1:**
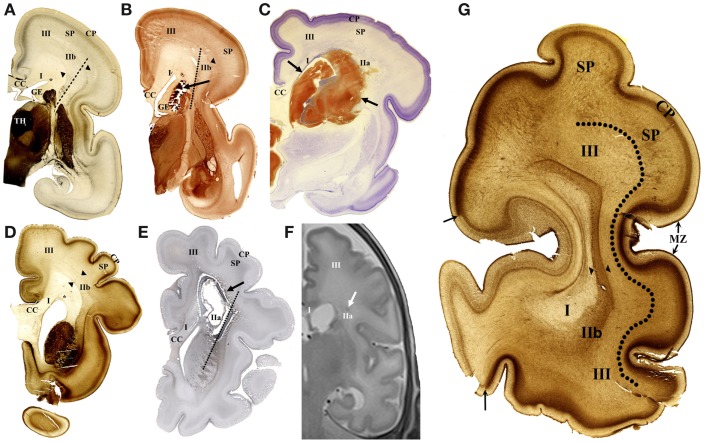
**Cellular compartments in the cerebral wall of the preterm brain and typical lesions shown on coronal sections**. The acetylcholinesterase (AChE) histochemistry **(A,B,D,G)**, Nissl-stained section **(C)**, immunohistochemical fibrillar staining **(E)**, and *in vivo* MR imaging **(F)** in early preterm of 26 PCW **(A–C)** and late preterm of 35 PCW **(D–G)**. In early preterm, deep periventricular compartment consists of ventricular–subventricular zone, ganglionic eminence (GE), and white matter segment (WMS I). Intermediate compartment contains crossroads (WMS IIa), sagittal strata (WMS IIb – between arrowheads), and centrum semiovale (WMS III). Typical small hemorrhagic lesion is visible in periventricular segment I **(B)**. Large hemorrhagic lesion [between arrows in **(C)**] destroys WMS I and IIa. In late preterm, cystic lesion is shown extending throughout WMS I and IIa **(E)**. Small hemorrhagic lesion is located in WMS IIa on *in vivo* MR image **(F)**. AChE histochemistry shows main compartments and WM segments on coronal section through occipital lobe **(G)**. Periventricular compartment, including WMS I, is followed in radial direction with sagittal strata (WMS IIb, between arrowheads). Next compartment, centrum semiovale (III) is delineated from subplate (SP) with broken line. Arrows indicate border between primary visual cortex (light staining) and area 18 (heavy staining). TH, thalamus; SP, subplate; CC, corpus callosum; MZ, marginal zone; asterisk, PVP pathway zone; broken lines **(A,B,E)** indicate axis of internal capsule.

1. Deep (periventricular) compartment includes proliferative fetal zones (ventricular–subventricular zone and ganglionic eminence) and adjacent periventricular fiber systems (65). This compartment roughly corresponds to white matter segment I of Von Monakow (11, 18) (Figure 1). Periventricular compartment contains massive callosal system and periventricular fiber system (segment I), consisting of associative fronto-occipital fascicle (FOF), cortico-striatal fibers within the subcallosal bundle and fronto-pontine pathways (8) (see Table 2).
2. Intermediate compartment (intermediate zone of mid-fetal period) contains crossroads of projection pathways (segment IIa) (11), their growth trajectories within sagittal axon strata (segment IIb) (8, 18) and centrum semiovale (segment III), which develops in late preterms (Figures 1A,D,G). Intermediate compartment contains major main telencephalic fiber systems (12, 25, 26, 54, 74). Crossroads are composed of massive projection fibers in the root of corona radiata (with thalamocortical and cortico-fugal radiating fibers), which are crossed by callosal radiation and the deepest associative sagittal fibers, surrounded by large amount of ECM (11). Sagittal strata (segment IIb) are most prominent in the occipital lobe and contain projections from sensory thalamus, projection from associative thalamus (pulvinar) and capsula externa radiation with basal forebrain cholinergic afferents and cortico-cortical associative fibers (15, 16). The centrum semiovale (segment III) (75) is composed of massive long associative fiber systems and projection fibers (see Table 2).
3. Superficial compartment is composed of three transient zones constituting the neocortical anlage: the subplate, the cortical plate, and the marginal zone (Figures 1D,G). During the late gestation and perinatal period, the gyral white matter (segment IV) develops in the superficial compartment. The subplate is fibrillar, deep portion of the cortical anlage, containing different cellular elements: postmigratory neurons with early functional activity, early formed synapses, axonal plexus of “waiting” afferent fibers, migratory neurons, different glial cell lines, and large amount of ECM (23). MRI properties of the subplate (high intensity on T2 and low intensity on T1 sequences) are mainly caused by large extra-cellular space, hydrophilic ECM, and anisotropic structure. The gyral white matter (segment IV) and intracortical fibers (segment V) are poorly developed in preterm brain. Superficial compartment is dominated by the subplate zone [not defined by Von Monakow; for delineation criteria see Ref. (54)]. Before 26 PCW, the subplate contains “waiting” afferent fibers from thalamus arranged in fibrillar network, and in later preterm period (after 28 PCW) growing front of the most superficial associative fibers. The gyral white matter (segment IV) develops during the late gestation and parallel with resolution of the subplate becomes closely adjacent to the cortical plate. However, in the neonatal brain, the subplate remnant still exists and serves as a growth zone for short cortico-cortical fibers and U-fibers (66).

The main differences between the cerebral wall of late versus early preterms were described recently (66). For the purpose of this study, it is important to note the following differences: an enlargement of the centrum semiovale, the formation of sulci with reduction of the subplate (Figure 1G), and thickening of the corpus callosum (callosal plate).

## Results

Using the above described spatial (topographical) and temporal (developmental age) criteria, we will describe: (a) typical lesions and their laminar landmarks in preterm infants, (b) morphological types and radial extent of MR abnormalities in preterms at term age, and (c) structural longitudinal *in vivo* MRI changes after the first year of life in the same group of patients who were scanned at term age.

### Typical lesions in preterm infants and its laminar landmarks

#### Lesions in the periventricular compartment – periventricular pathway zone

Periventricular lesions occupy area medial to the radiation of internal capsule (Figure 1, dotted line). Two types of lesion were seen to be restricted to the zone of periventricular pathways. The first is acute, localized periventricular hemorrhage in the space between the ganglionic eminence and the periventricular pathway zone (PVP) zone (Figure 1B). The ganglionic eminence is the most prominent, cell-dense periventricular structure in the preterm brain (Figures 1A,B). The PVP pathway zone is triangular area situated at the lateral angle of lateral ventricle (Figures 1A,D). The second type of lesion is cystic formation (with cavity) situated in the PVP pathway zone (segment I). Larger hemorrhagic lesions extend to the exit of the internal capsule (Figure 1C). This type of lesion affects all periventricular pathways [the subcallosal fascicle with cortico-striatal fibers, the fronto-pontine motor pathway, and the most massive FOF (65)] and extends into the intermediate compartment (segment II, see Table 2).

#### Lesions of intermediate compartment

Larger cystic lesions (Figure 1E) also involve the segment II that is periventricular crossroads of pathways (11). The presence of thick fibrillar–glial capsule around the cystic lesion indicated early development of this lesion. MR imaging offers a unique opportunity to visualize small lesions in the periventricular crossroads (Figure 1F).

We conclude that these periventricular focal lesions in preterm infant damage segment I of white matter (PVP) and segment IIa (crossroads) and cause MR abnormalities, leaving intact the subplate zone/centrum semiovale and gyral white matter (in later preterms). Lesions of the occipital crossroads are accompanied with lesions of the root of radiation of the posterior limb of the internal capsule (PLIC) and may also affect occipital sagittal strata. The lesions of periventricular and crossroad segments also damage proliferative and migratory zones within the cerebral wall.

As stated above, the centrum semiovale is situated between sagittal strata and the subplate, and was developed only in later preterms (Figure 1G). The most common finding (“abnormality”) is an increase in MRI T2 signal intensity (73). This developmental abnormality corresponds topographically to the definition of diffuse periventricular leukomalacia (31).

We found that decrease in visibility of anatomical border between sagittal strata and centrum semiovale – subplate, may be more indicative of the prospective acute lesion of centrum semiovale than the change in MRI signal intensity alone (Figure 1F). The external capsule radiation is the only reliable landmark (54) for anatomical delineation between focal periventricular lesions and diffuse lesions in the centrum semiovale (73). These landmarks can be easily determined only in the frontal and occipital lobe (Figure 1G).

#### Lesions of superficial compartment

In this study, lesions of the subplate and the cortical plate have not been described convincingly at MRI level. However, at the histological level, there is evidence for significant reactivity of astroglia in the deep portion of subplate after hypoxic-ischemic lesions (76) and widespread (although non-specific) changes of subplate neurons (52).

### Morphological types and radial extent of MRI abnormalities in preterm children at term age in reference to white matter segments and the subplate remnant

**Figure 2:**
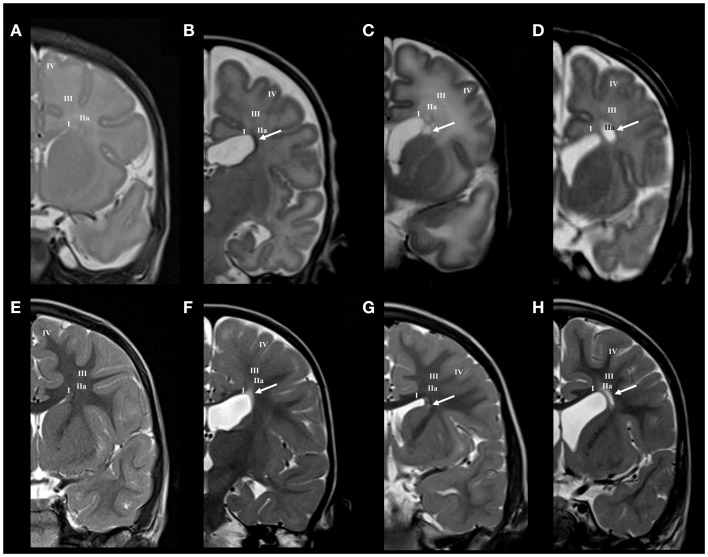
**Longitudinal MRI follow-up of perinatal periventricular pathways lesions on coronal T2 images**. Normal findings of periventricular white matter at term **(A)** and at the age of 3 years **(E)**. Early preterm [born at 25 PCW, birth weight (BW) = 800 g, SNAPII = 35, SNAPPEII = 63, Apgar score (AS) = 9/10] with hemorrhagic lesion in area of subcallosal fascicle at term equivalent age [arrow in **(B)**], which changed to gliotic scar at the age of 2 years [arrow in **(F)**]. Early preterm (born at 28 PCW, BW = 1080 g, SNAPII = 13, SNAPPEII = 31, AS = 2/4) with cystic lesion in area of subcallosal and fronto-occipital fascicle at term equivalent age [arrow in **(C)**], which changed to discrete scar within fronto-occipital fascicle at the age of 3 years [arrow in **(G)**]. Late preterm (born at 31 PCW, BW = 1670 g, SNAPII = 8, SNAPPEII = 8, AS = 8/10) with large cystic lesion at the crossroad area at term equivalent age [arrow in **(D)**], which appears as smaller cystic lesion at the age of 3 years [arrow in **(H)**]. Numbers I–IV represent segments of white matter as previously described in text.

The neonatal brain at term age shows well developed deep and intermediate segments of white matter (segments I, II, and III), while distal segment (gyral white matter) is still developing. The subplate is reduced in thickness and is described as the subplate remnant (66). The neocortex is fully laminated, but appears very immature due to the higher packing density of its neurons. The white matter segment V (radii), which consist of bundles of radially arranged axons, is poorly developed. In preterm infants at term, the crossroads, centrum semiovale and developing gyral white matter show, in higher percentage than normal term brain, an increase in MRI signal intensity (Figure 2).

#### Lesion of periventricular compartment

Small hemorrhagic lesions (five cases) were seen to occupy territory of Muratoff’s subcallosal fascicle in the PVP, lateral to the angle of the ventricle (Figure 2B). Periventricular cystic lesions (four cases) destroy, in addition to subcallosal fascicle, more laterally positioned FOF (Figure 2C).

#### Lesion of intermediate compartment

**Figure 3:**
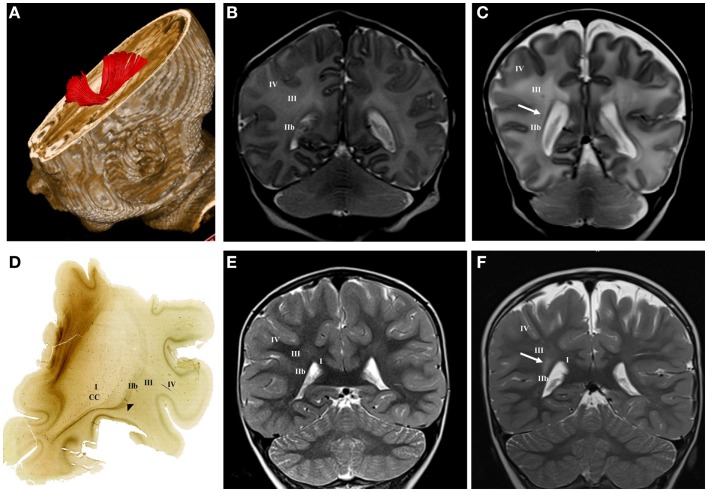
**DTI tractography reconstruction of sagittal strata at term age (A)**. The acetylcholinesterase (AChE) histochemistry shows external sagittal stratum [arrowhead in **(D)**] at the frontal level at term age. Normal MRI findings of sagittal strata at term age **(B)** and at the age of 3 years **(E)** on T2 coronal images. Late preterm child (born at 30 PCW, BW = 1510 g, SNAPII = 16, SNAPPEII = 34, AS = 2/5) with punctate hemorrhagic lesion within sagittal strata at term equivalent age [arrow in **(C)**] and hyper-intensive lesion of sagittal strata at the age of 3 years [arrow in **(F)**] on T2 coronal images. Numbers I–IV represent segments of white matter as previously described, CC, corpus callosum.

**Figure 4:**
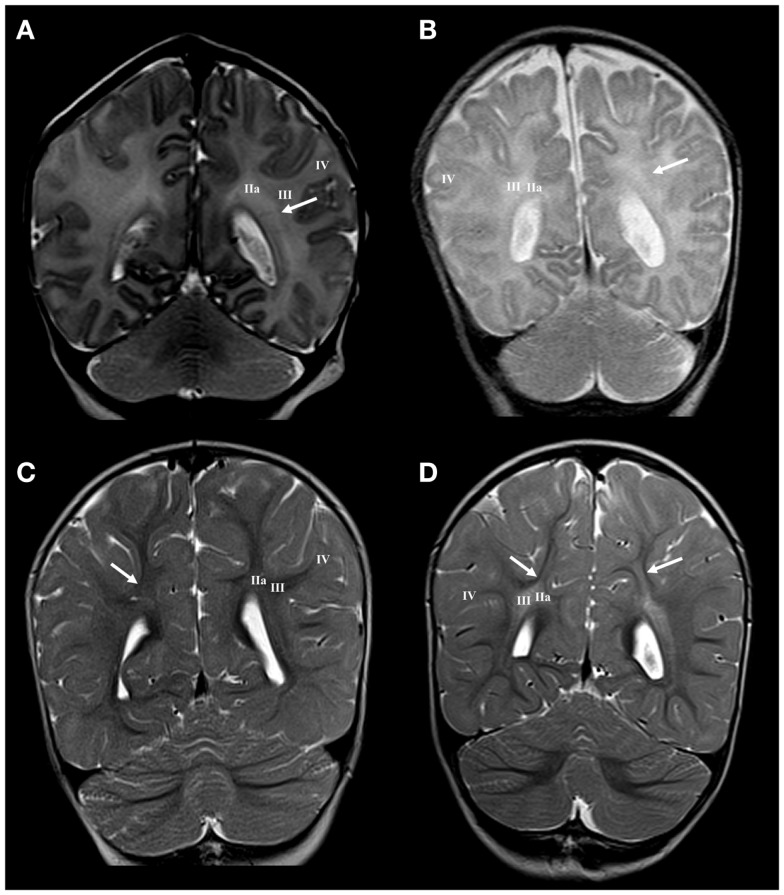
**Longitudinal MRI follow-up of centrum semiovale perinatal lesion on coronal T2 images**. Normal findings of centrum semiovale at term age **(A)** with visible border between parietal crossroad and centrum semiovale [arrow in **(A)**]. Normal findings at the age of 13 months with barely visible border between U-fibers and centrum semiovale [arrow in **(C)**]. Term born child (BW = 3960 g, SNAPII = 40, SNAPPEII = 58, AS = 0/4) with perinatal asphyxia and diffuse hyperintensity of white matter with diminished border between parietal crossroad and centrum semiovale at term equivalent age [arrow in **(B)**] but with enhanced visibility of border between U-fibers and hyper-intensive centrum semiovale at the age of 13 months [arrows in **(D)**]. Numbers I–IV represent segments of white matter as previously described.

Larger periventricular cysts stretch to the exit of internal capsule/root of corona radiata (Figure 2D) and involve crossroads of pathways (segment IIa). Lesions in the territory of sagittal strata are characterized by decrease in the visibility of borders between individual sagittal strata and the border between external sagittal stratum and centrum semiovale (Figure 3C). The most common MRI abnormality of centrum semiovale is an increase in T2 MRI signal intensity (Figure 4B). The characteristic abnormality is decrease in visibility of border between intermediate compartment (centrum semiovale and sagittal strata) and superficial compartment (gyral white matter and subplate remnant). This abnormality is prospective MRI structural evolution of the so-called diffused PVL.

#### Lesion of the superficial compartment

**Figure 5:**
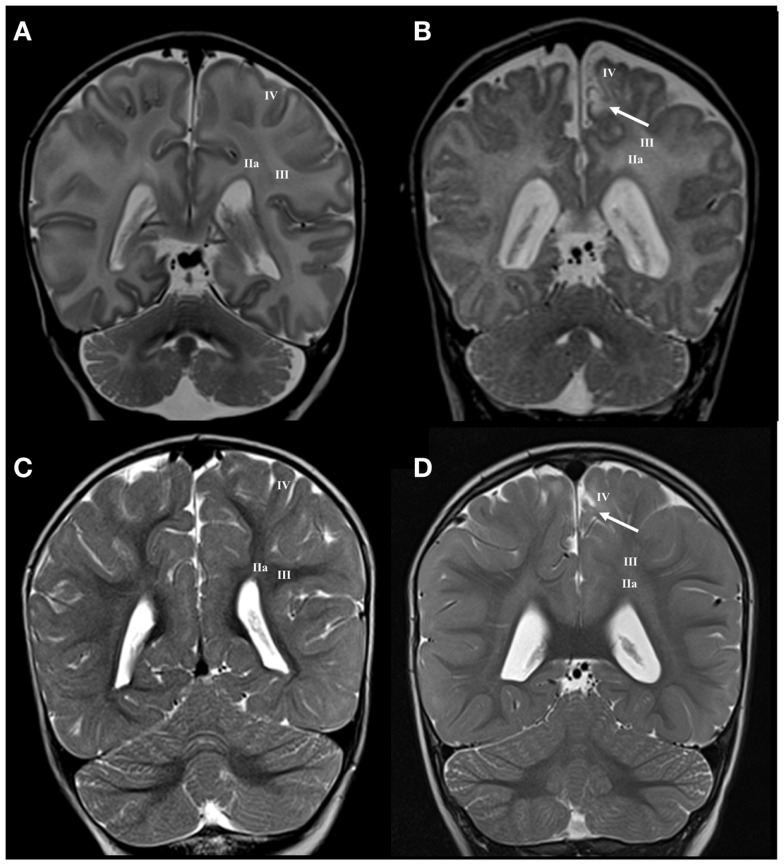
**Longitudinal MRI follow-up of perinatal gyral white matter and cerebral cortex lesion on coronal T2 images**. Normal findings of gyral white matter and cortex at term age **(A)** and at the age of 13 months **(C)**. Term born child (BW = 3220 g, SNAPII = 31, SNAPEII = 49, AS = 1/3) with perinatal asphyxia and hyperintensity of centrum semiovale and gyral white matter accompanied with cortical lesion at term equivalent age [arrow in **(B)**] and with hyper-intensive lesion of gyral white matter and cortex at the age of 13 months [arrow in **(D)**]. Numbers I–IV represent segments of white matter as previously described.

The presence of the subplate remnant, defined as narrow ECM rich transitional zone between the gyral white matter and the cortical plate, is marker of normal cortical organization at birth (66). Accordingly, the absence of delineation of the subplate remnant or sharp (enhanced) delineation (Figure 4) is a marker of possible lesion of distal (superficial) compartment of the cerebral wall during the late preterm period. The close spatial and developmental relationships between the subplate remnant and underlying gyral white matter make MRI delineation of these two sub-compartments extremely difficult. The presence of these transient sub-compartments may be demonstrated using different cellular, extra-cellular, and fibrillar markers at fine histological level (66). However, if there are significant regional changes in MRI signal intensity of the gyral white matter (Figure 5B), with loss of borders, one should consider the existence of abnormality of these two superficial sub-compartments. This can be confirmed by longitudinal imaging after the first year of life.

### Structural (longitudinal) *in vivo* MR changes of white matter and cellular compartments after the first year of life

The longitudinal developmental changes of cerebral compartments were analyzed in the same cohort of patients, which was described at term age.

#### Periventricular compartment

As expected, small hemorrhagic lesions in PVP area transformed in focal well delineated “scar-like” formation of increased T2 MRI signal intensity (Figure 2F). Small cystic lesions of periventricular compartment disappear. Instead, the area of periventricular pathways shows mild shrinkage with slight reduction of FOF (Figure 2G). Abnormalities of signal intensity and “scars” in the periventricular compartment may be confluent along the entire dorsal ventricular system.

#### Intermediate compartment

Large cystic lesions collapsed, but remained visible in the territory of crossroads and periventricular zone (Figure 2H). Semi-oval cystic formation may extend along the entire cerebral wall. After the first year, the distal portion of intermediate compartment (segment II), that is sagittal strata, shows loss of characteristic three-band appearance with abnormal signal elongated in the sagittal plane (Figure 3F). Abnormalities of MRI signal intensity, observed in PLIC area at term, later show narrower distance between sides of triangular crossroad area at the point where external and internal capsule continue in external and internal sagittal stratum. The most interesting finding in the intermediate compartment and distal compartment is enhanced difference between MRI signal intensity of centrum semiovale and subplate remnant (Figure 4D).

#### Superficial compartment

In cases with regional gyral white matter lesions, there is selective MRI signal abnormality with loss of border with adjacent compartments. In these cases, the centrum semiovale shows normal MRI signal intensity, which indicates a proper myelination process (Figure 5).

In conclusion, the analysis of cerebral compartments and white matter segments after the first year shows, in some cases, that an increased MRI T2 signal intensity observed at term did develop in a characteristic MRI abnormality: the sharp delineation of the subplate remnant and U-fiber zone and MRI signal abnormalities remain present in the centrum semiovale. This indicated selective vulnerability of main body of associative fibers in the centrum semiovale.

## Discussion

We have reviewed the evidence on organization and developmental dynamics of cellular compartments in the cerebral wall in the third trimester of human gestation and illustrated how precise anatomical landmarks can be used for description of radial extent of lesion on histological sections and conventional MR images. In addition, we evaluated the significance of borders between cerebral compartments for MRI analysis of abnormalities of premature infant brain at term equivalent age and documented their structural reorganization in longitudinal (second MR) imaging. The concept of transient cellular compartments as a crucial spatial framework for analysis of neurogenetic events in the developing cerebral cortex has been elaborated for decades since the beginning of modern era of developmental neurobiology (9, 13, 19, 22). There is a general agreement that dynamic changes of transient compartments reflect basic pattern of histogenesis of the developing cerebral cortex (9, 19, 54, 77–79). Laminar organization of fetal cerebral compartments was useful for description of developmental changes of the cerebral cortex in current imaging studies (12, 24–26, 28, 54, 66, 80).

Due to the fact that neurogenetic events take place in specific, developmentally important cerebral compartments, the laminar extent of cerebral lesion may help to understand developmental disturbances after hypoxia ischemia and hemorrhagic lesion in the third trimester of gestation and equivalent preterm period (30, 34, 53, 62). In the present paper, we have extended the conceptual framework of transient compartments to the concept of radial vulnerability of different white matter segments and compartments in the cerebral wall. The distribution of different classes of well-known cortical projection, commissural and associative pathways arranged radially (from ventricle to pia) within different spatial compartments, may be easily related to classical description of focal periventricular and diffused lesions in preterm brain (30, 31, 53, 61, 63, 64).

**Figure 6:**
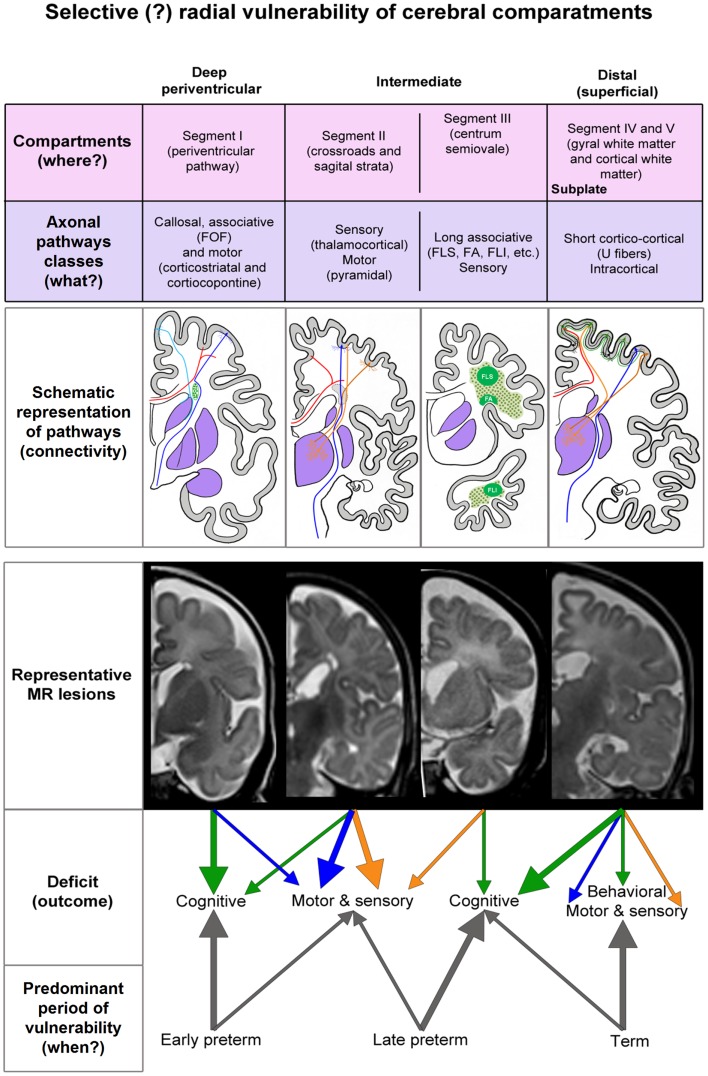
**Summary diagram of the concept of radial vulnerability of cerebral compartments, which are listed from ventricle (left) to pia (right)**. Schematic representation of pathways is constructed on the basis of our previous studies (references in the text). MR representative lesions are selected from clinical cases other then presented in Figures 1–5. The proposed deficit (arrows) is derived from the prospective function of lesioned classes of axonal pathways. FOF, fronto-occipital fascicle; FLS, longitudinal superior fascicle; FA, arcuate fascicle; FLI, longitudinal inferior fascicle.

We found that both focal and diffused lesions, depending on their radial extent, affect well identified and spatially segregated classes of axons: periventricular pathways of mixed modalities within ventricular/subventricular zone (65), major projection pathways within the crossroads (11, 54) and sagittal strata (18, 54, 75, 81), associative pathways within the centrum semiovale and deep cortical subplate and thalamocorticals in the subplate of early preterms. Moreover, characteristic spatial arrangement of these axonal pathways shows time-related sequential growth, with some overlap during 22–36 PCW. These two parameters, spatial (compartments) and developmental (periods), are two factors, which determine the extent and nature of lesions of white matter pathways, the subplate and incorporated nerve cells (31, 82), resulting in dynamic picture of vulnerability across the cerebral wall. By analyzing MR images in small cohort of premature infants at birth and after the first year of life, as well as histological sections of selected post-mortem cases, we found that there are well delineated lesions, which almost selectively damage early differentiating associative FOF and motor pathways in the periventricular zone situated medial to the root of the corona radiata. This periventricular focal lesion may be easily followed from term equivalent age to the second MRI scan after the first year of life. The prospective deficit after this type of lesion is not well defined, but it may include general cognition due to the damage of the robust FOF and impairment of motor functions related to the damage of fronto-pontine and cortico-striatal pathways (Figure 6). The lesions in this medial periventricular area are complicated by the fact that the very same lesion may damage cell proliferation in the ganglionic eminence and migration of GABAergic neurons from the ganglionic eminence (8). The most complex and difficult to interpret may be lesions of periventricular crossroads of pathways (11). Kidokoro and Inder (64) have shown that visibility of crossroads is a sign of normal development while poor delineation combined with increase in MRI signal intensity is related to poor neurodevelopmental outcome. We confirmed this finding, but also extend this observation to the sagittal strata: we demonstrated that decrease in visibility and delineation of three sagittal strata (internal, central, and external) (8) in the frontal and occipital lobe are important signs of MRI abnormality. These MRI abnormalities probably indicate the lesions of thalamocortical sensory pathways and pathways from the associative thalamic nuclei, such as pulvinar, which are the most voluminous contingent of intermediate sagittal stratum (18, 54). In our previous studies, we emphasized that during the period between 24 and 26 PCW thalamic fibers from mediodorsal nucleus and pulvinar complex accumulate below the cortical plate and penetrate frontal and parietal associative cortex (3, 15, 16, 82).

Thus, loss of “normal” border between external sagittal stratum and centrum semiovale is indicative of lesion of thalamocortical pathways and may be accompanied with thalamic lesions (31, 42, 73, 83, 84).

One of the most interesting observations in our material is lesioning of centrum semiovale with preservation of the subplate remnant. This developmental lesion results in loss of border at term, but shows “enhanced” delineation of subplate remnant from centrum semiovale (segment III) during the subsequent development. This reorganization of borders between cerebral compartments requires developmental interpretation of subplate zone, centrum semiovale, related thalamocortical and associative pathways.

The centrum semiovale develops in the late preterm, between 32 and 36 PCW, when thalamocortical fibers have already relocated from the subplate zone (4, 10, 19). The centrum semiovale and the deep subplate serve as main compartments for growth of long associative cortico-cortical fibers. Some of these growing pathways may be damaged by diffuse distal hypoxic ischemic lesions (31). As a consequence, centrum semiovale shows signal abnormality at birth. After the first year, impairment of myelination and other unexplored pathological (developmental?) changes may cause abnormal MRI signal. On the other hand, short cortico-cortical fibers (including U-fibers) grow through the subplate remnant during a different developmental period that is during the early postnatal life. Therefore, they are not damaged during the preterm period (like long cortico-cortical fibers) and show “normal” MRI signal intensity during the postnatal period. Thus, we interpret the phenomenon of enhanced border between the subplate remnant and the centrum semiovale as a consequence of differential period of vulnerability for these two compartments.

The lesion of subplate neurons is the most enigmatic. There is no doubt that subplate neurons are vulnerable during preterm period and form a neuronal substrate in Volpe’s “complex amalgam of destructive and developmental disturbances.” This prediction was confirmed in neuropathological studies after hypoxic ischemic lesion (52). Considering complex developmental role of subplate zone and subplate neurons (10, 23, 85–87) damage of subplate neurons may have multiple effects on cortical development. From developmental studies (23, 88), it can be predicted that lesion of subplate neurons and other subplate cellular and extra-cellular elements will interfere with growth of thalamocortical fibers. As stated above, we believe that the growth-related vulnerability of associative cortico-cortical pathways in this distal segment of white matter (centrum semiovale) at the interface with subplate occurs later then vulnerability of deeper thalamocortical fibers containing segments of fetal white matter (sagittal strata and crossroads including PLIC). If our prediction is correct, then vulnerability of thalamocortical projection axonal pathways occurs between 22 and 28 PCW that is in early preterm period, when these fibers wait and accumulate in subplate and penetrate cortical plate.

The mechanisms that contribute to the pathology of axonal pathways in preterm brain are poorly understood. It is also not clear whether, when and how growing axons are primary target for hypoxia ischemia or their lesion occurs secondary to the damage of their cell bodies of origin, other connected neurons, such as subplate neurons or glia cells (31, 53).

Haynes et al. (89) have shown that damage of axons may be significant component of diffuse PVL and can be detected by the apoptotic marker fractine. The axons may lose their integrity because they fail to find their path due to the hypoxic ischemic disturbance of ECM or death of cells, which secrete axonal guidance molecules. The axonal guidance is event with complex interactions of receptor molecules and chemo-attractant or chemo-repellant molecules, which are present and expressed in characteristic gradients within the cerebral wall (5, 90).

This complex axonal guidance system is the main mechanism in the complex process of path-finding and target selection (2, 5–7, 19). The distribution of large amount of ECM in vulnerable compartments such as periventricular crossroads and subplate, lead us to propose that one of the most important basic mechanisms in periventricular white matter injury and encephalopathy of prematurity (53) is abnormality in organization and content of ECM and impaired synthesis of axonal guidance molecules (11, 66).

Although it is not known which cells produce ECM molecules such as chondroitin sulfate, containing glycosaminoglycans, it is very likely that these cells reside along axonal growth trajectories and in the subplate. It is logical to assume that cytotoxic substances produced during hypoxic ischemic events damage not only pre-oligodendrocytes (53, 91) but also affect astrocytes (31, 76). Astrocytes are distributed within all compartments of cerebral wall during the third trimester of gestation and are candidates for different metabolic and synaptic functions, including the synthesis of ECM. It is interesting that astrocyte activation is more characteristic of late preterm period (76) and also involves subplate astroglia without macroscopic changes (31, 76, 92). Recent studies of white matter injury in preterm brain suggest that damage of pre-oligodendrocytes place important role in the pathogenesis of prenatal hypoxia (48), especially in the diffuse component of periventricular leukomalacia (93). This view is consistent with opinion that impaired myelination is important factor in axonal deficit and contributes to the decrease in white matter volume in prematurely born infants (46). Since centrum semiovale with massive associative pathways is involved in diffuse non-cystic white matter injury, hypoxic ischemic lesions of pre-oligodendrocytes may contribute to the changes observed in this compartment in our material. Immunocytochemical preparations for myelin basic protein (MBP) of post-mortem brains of children who died with evidence of hypoxic-ischemic episodes show preserved myelination of projection fibers but poor myelination of centrum semiovale (65). This corresponds to our MRI findings. One of the most important conclusions from the present paper is that the time of injury and radial extent of lesion from ventricle to pia have effects on subsequent organization of white matter and cortex. This is in agreement with the concept that encephalopathy of prematurity is an amalgam of destructive and developmental disturbances (31). Our data relevant to this concept show that, when imaging and histological data are presented for all compartments and segments of white matter, from ventricle to pia (66), and for borders between compartments and changes in MRI signal intensity (63, 64, 72) clear spatial relationships with histological landmarks can be detected (54, 66).

The concept of radial vulnerability and data presented in this paper are limited due to the fact that we did not analyze connected subcortical structures (caudate, putamen, thalamus, amygdala, cerebellum, brain stem nuclei, and spinal cord). This will be subject of our future studies.

## Conclusion

In conclusion, developmental vulnerability changes along radial axis in relation to growing axonal strata and deep to superficial differentiation of neurons in the subplate and cortical plate. Deep, periventricular lesions (PVH and focal PVL) damage fronto-occipital associative, cortico-striatal, and corticopontine projection pathways and will result in cognitive and motor deficit (Figure 6). Periventricular lesions also interfere with proliferation and migration, which contributes to the complexity of the lesion. Lesions within the internal capsule, crossroads, and sagittal strata damage predominantly projection pathways (sensory and motor), with possible cognitive component. Vulnerability of thalamocortical pathways within the crossroad and sagittal strata seems to be characteristic for early preterms, while vulnerability of association pathways in the centrum semiovale seems to be predominant feature of late preterms. The cerebral compartments, which are not affected in the preterm brain, the superficial subplate, and the cortical plate with short cortico-cortical fibers, are important substrate for later developmental plasticity and functional recovery of the damaged infant brain. However, if damaged during prenatal period, the subplate zone, subplate cells, cortical cells, and short cortico-cortical connections will cause cortical type of deficit and combination of behavioral, motor, sensory, and cognitive components. The delineation between different, intermediate, and superficial segments, segments of white matter (external sagittal stratum, centrum semiovale, and gyral white matter) subplate remnant and changes in signal intensity together with radial extent of MRI abnormalities seem to be important indicators of lesions of association pathways in prematurely born infants at term. In contrast, short cortico-cortical and U-fibers seem to be intact due to the late developmental schedule. Our study revealed that analysis of radial extent and laminar delineation of MRI abnormalities in the cerebral compartments may indicate lesion of different classes of axonal pathways and may help in prediction of structural and functional outcome after prenatal and perinatal lesions.

## Author Contributions

All authors have participated equally in the work and have full access to all the data in the study and take responsibility for the integrity of the data and the accuracy of the data analysis. Ivica Kostović has proposed basic concept of cerebral compartments, corridors of axonal growth, and radial vulnerability. Vesna Benjak and Mirna Kostović-Srzentić have performed clinical testing of preterm children and Nataša Jovanov-Milošević has performed histochemical and immunohistochemical staining of post-mortem brain. Milan Radoš has performed all *in vivo* MR scans. All authors (Ivica Kostović, Vesna Benjak, Mirna Kostović-Srzentić, Nataša Jovanov-Milošević, and Milan Radoš) have participated equally in interpretation of data, article writing, and approval of final version.

## Conflict of Interest Statement

The authors declare that the research was conducted in the absence of any commercial or financial relationships that could be construed as a potential conflict of interest.
